# Transition from a uni- to a bimodal interfacial charge distribution in $\hbox {LaAlO}_3$/$\hbox {SrTiO}_3$ upon cooling

**DOI:** 10.1038/s41598-020-74364-7

**Published:** 2020-10-27

**Authors:** M. Zwiebler, E. Di Gennaro, J. E. Hamann-Borrero, T. Ritschel, R. J. Green, G. A. Sawatzky, E. Schierle, E. Weschke, A. Leo, F. Miletto Granozio, J. Geck

**Affiliations:** 1 Institut für Festkörper- und Materialphysik, Technische Universität Dresden, 01062 Dresden, Germany; 2 Dipartimento di Fisica “E. Pancini”, Università di Napoli “Federico II”, Complesso Universitario di Monte S. Angelo, Via Cintia, 80126 Naples, Italy; 3 CNR-SPIN, Complesso Universitario di Monte S. Angelo, Via Cintia, 80126 Naples, Italy; 4 Stewart Blusson Quantum Matter Institute, University of British Columbia, Vancouver, V6T 1Z1 Canada; 5 Department of Physics and Engineering Physics, University of Saskatchewan, Saskatoon, S7N 5E2 Canada; 6 Helmholtz-Zentrum Berlin, BESSY, Albert-Einstein-Str. 15, 12489 Berlin, Germany; 7 Dipartimento di Fisica “E. R. Caianiello”, Università degli Studi di Salerno, Fisciano, Italy; 8 CNR-SPIN, Campus di Fisciano-Salerno, Fisciano, Italy; 9 Würzburg-Dresden Cluster of Excellence ct.qmat, Technische Universität Dresden, 01062 Dresden, Germany

**Keywords:** Electronic properties and materials, Surfaces, interfaces and thin films

## Abstract

We present a combined resonant soft X-ray reflectivity and electric transport study of $\hbox {LaAlO}_3$/$\hbox {SrTiO}_3$ field effect devices. The depth profiles with atomic layer resolution that are obtained from the resonant reflectivity reveal a pronounced temperature dependence of the two-dimensional electron liquid at the $\hbox {LaAlO}_3$/$\hbox {SrTiO}_3$ interface. At room temperature the corresponding electrons are located close to the interface, extending down to 4 unit cells into the $\hbox {SrTiO}_3$ substrate. Upon cooling, however, these interface electrons assume a bimodal depth distribution: They spread out deeper into the $\hbox {SrTiO}_3$ and split into two distinct parts, namely one close to the interface with a thickness of about 4 unit cells and another centered around 9 unit cells from the interface. The results are consistent with theoretical predictions based on oxygen vacancies at the surface of the $\hbox {LaAlO}_3$ film and support the notion of a complex interplay between structural and electronic degrees of freedom.

## Introduction

The interfaces between different transition metal oxides provide an exciting arena for novel electronic quantum systems confined into a planar, almost two-dimensional geometry. Especially the so-called two-dimensional electron liquid (2DEL) at the interface between insulating LaAlO$_3$ (LAO) and SrTiO$_3$ (STO) has been studied intensively in this regard. The interest in this system follows the observation of an interfacial metallic 2DEL formed when a film of polar LAO, exceeding a critical thickness of 4 unit cells, is grown on a (001)-oriented, TiO$_2$-terminated $\hbox {SrTiO}_3$ substrate^1^. Later this effect was also observed for other polar band insulators such as LaGaO$_3$^2^, NdGaO$_3$^3^, NdAlO$_3$ and PrAlO$_3$^4^ on STO, for which the 2DEL is robust even if exposed to highly oxidising conditions during or after growth. This 2DEL exhibits a variety of fascinating effects like electric field-tunable superconductivity^5,6^, a large and also electric field-tunable Rashba coupling^7^, an exceptionally large spin-to-charge conversion efficiency^8^ and possibly unconventional magnetism^9^.

Thiel *et al.* first demonstrated a strong field effect at low temperatures, in which the 2DEL conductivity strongly depends on the back gate voltage (V$_G$), i.e., the voltage difference between the 2DEL and the back of the substrate^10^. The field effect is due to a transfer of charge between the gate electrode and the conducting channel, i.e. a charge redistribution within a field effect device (FED). It has been argued previously that this redistribution involves a changing population of localized and mobile electronic states within the LAO/STO-heterostructure^11–13^. In addition, this charge distribution has been found to be temperature dependent as well^14–17^.

The atomic scale depth profile of the 2DEL at different gate voltages and temperatures is therefore a key feature needed to interpret the transport properties of these FEDs. But, to our knowledge, such a high-resolution depth profile has not yet been reported. This is exactly the motivation of the present study, where we combine electric transport and resonant X-ray reflectivity (RXR) measurements into one experiment and use this to explore the electronic depth profile in a LAO/STO heterostructure as a function of the applied back-gate voltage and temperature. In this way we aim at clarifying the relation between the macroscopic charge transport of our device and the microscopic depth distribution of the 2DEL.

**Figure 1:**
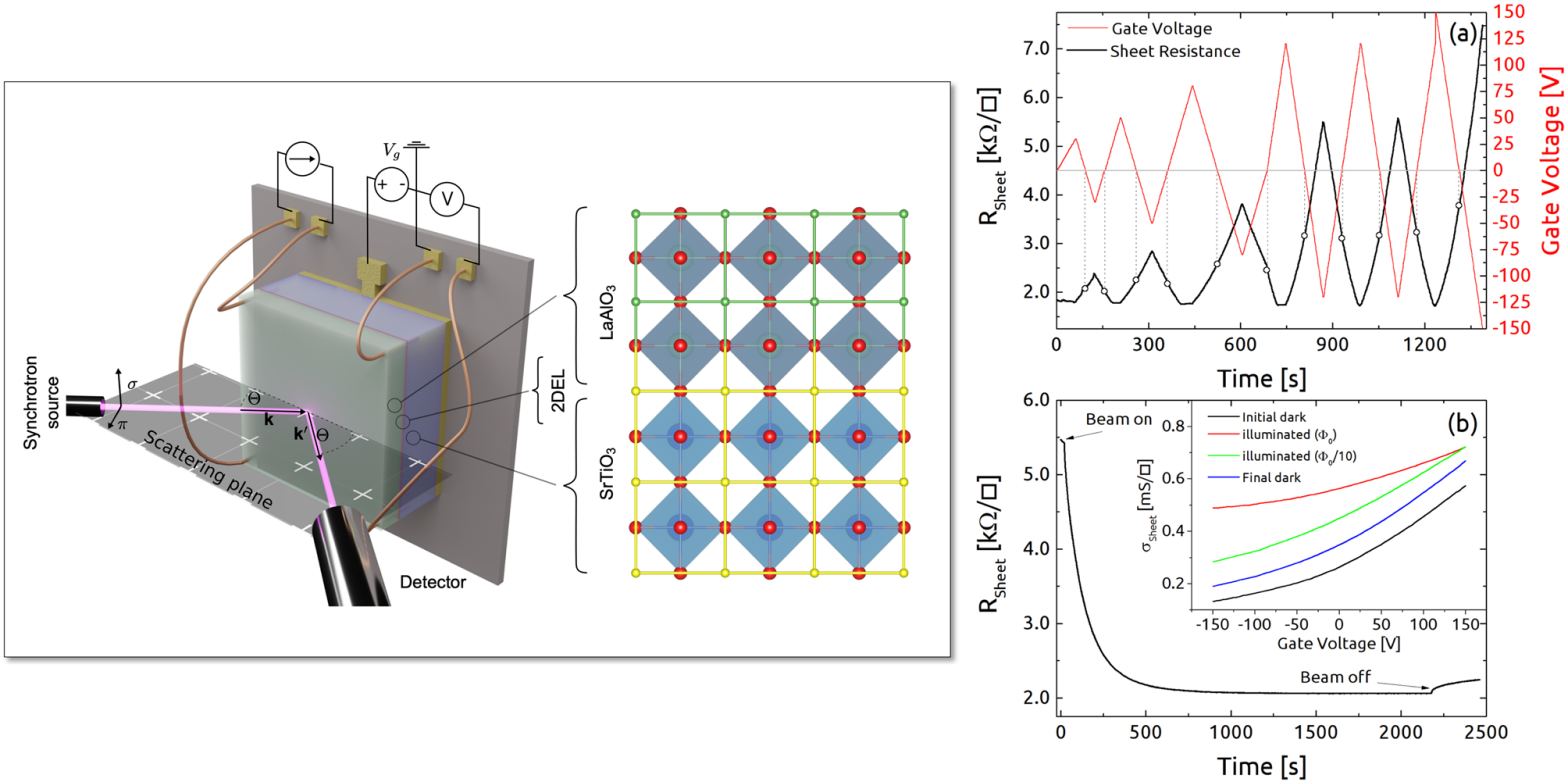
Left: Illustration of the FED-setup for the conductivity measurement during RXR and XAS measurements. The measurements were performed in a 4-point van der Pauw-setup in constant current mode at $\pm 10\mu$A. Right: (**a**) FED-initialization at $T=11$ K. Cycling of gate voltage in dark (red curve) and the corresponding hysteretic response of the sheet resistance (black curve). Hollow dots highlight the non-reversible growth of $R_{sheet}$ at zero $V_G$ after every increased value of the positive voltage. (**b**) Effect of X-ray irradiation: Temporal evolution of the sheet resistance at $T=11$ K and $V_G=-150$ V under illumination with 490 eV X-rays. These data have been taken after a previous exposure to X-rays. Hence the initial sheet resistance differs from the initialization measurement in (**a**) done before X-ray exposure. This hysteretic effect can also be seen in the inset, where the influence of X-ray irradiation on the electrical conductivity versus V$_G$ is shown.

## Experiment

In order to induce controlled changes in the 2DEL via the field effect, a gate electrode at the back of the STO substrate was used as shown in Fig. 1. We resorted to 4-point sheet resistance measurements in van der Pauw configuration (cf. Fig. 1), performed in real time in the same experimental chamber where the RXR measurements were done. At each measurement point, the voltage was measured at sample currents of $\pm 10\, \upmu{\text{A}}$. In order to characterize the hysteretic behavior of the LAO/STO FED^6,11,18^ and to initialize it according to the commonly adopted approach, we applied the cycle shown in Fig. 1(a), while keeping the beam off and the temperature at 11 K.

RXR is very sensitive to spatial variations of electric properties at an interface^19^. By choosing the appropriate resonance (absorption edge), the valence, spin, or the orbital configuration of the resonant sites can be probed through the corresponding atomic scattering amplitudes $f(\omega )$. As a result, spatial modulations of these electronic features at an interface are clearly visible in RXR. The RXR measurements were done in three modes. The first one is the so-called fixed-$\Theta$ mode, where the incident angle $\Theta$ is fixed, while the photon energy is swept across the studied resonance. The second one is the fixed-*q* mode, which keeps the scattering vector (0,0,q) fixed while changing the photon energy. The third mode is the fixed-E mode. In this case the photon energy is fixed and the specular RXR is measured as a function of the incident angle $\Theta$, viz. as a function of *q*. Incident photon energies in the vicinity of the Ti $L_{2,3}$, the La $M_{4,5}$ edges and at a few off-resonant energies far away from any absorption edge of the sample were investigated. The collected data set consists of $\theta -2\theta$ scans with incident angles $\theta$ in the range $5^{\circ }<\theta <75^{\circ }$. RXR measurements at the Ti-edges were performed using linear $\sigma$ and $\pi$ polarization of the incident X-ray photons, whereas for all other energies only $\sigma$ polarization was used. $\pi$ ($\sigma$) designate a linear polarization parallel (perpendicular) to the scattering plane. In total we have collected 41 RXR curves per polarization around the Ti edges, 5 around the La edges with $\sigma$ polarization and 3 at off-resonant energies (i.e., 400 eV, 700 eV and 1000 eV). The RXR-measurement were done at room temperature and at 11K with a working gate voltage of $\pm 100$ V ($\pm 2$ kV/cm) for the field effect. Each data set for a given condition consists of approximately 12000 data points.

## Results

### X-ray irradiation effect

We initialized the sample by cycling the gate voltage $V_G$ as shown in Fig. 1(a) (red curve, right axis). The response of our sample to this initialization cycle is also reported in Fig. 1(a) (black curve, left axis), which agrees very well with previous reports^11,18^. The applied voltage cycle highlights the existence of a lower bound R$^{Min}_{sheet}$ = 1.8 $k\Omega /\square$ in the gate-dependent sheet resistance values. This value is achieved again and again during cycling, and precisely every time $V_G$ equals or exceeds the previous positive maximum value. Also this behavior agrees well with previous studies of LAO/STO FEDs, where the saturation at positive $V_G$ has been interpreted in terms of a completely filled interface potential well^11,18^. Another well-known and characteristic feature of the initialization is the non-reversible increase of the zero bias R$_{sheet}$ with cycling (Fig. 1(a), open circles). After the initialization, changing $V_G$ between $\pm 150$ V causes a linear response in the sheet resistance ($\Delta R_{sheet}/R_{sheet} \propto -\Delta V/V$) in agreement with the expectations for such a FED.

A dramatic effect on the 2DEL transport properties is found under irradiation with soft X-rays. After the sample was switched to the high-resistance state by $V_G=-150$ V it was exposed to the X-ray beam. As can be observed in Fig. 1(b), this exposure induced a rapid transition to a lower resistance state, with a sheet resistance very close to the one induced by a large positive $V_G$. While the resistance of the irradiated state in saturation, i.e. after long-time exposure, turned out to be affected only weakly upon further reducing $V_G$ to more *negative* values, the inset in Fig. 1(b) shows that the sample-averaged electrical transport properties, here expressed in terms of conductance, can still be manipulated by increasing $V_G$ towards *positive* values. The data in the inset show that the sample-averaged 2DEL properties probed in our transport measurements are both affected by X-ray irradiation and by back-gate voltage. The two effects appear to add-up in a large interval until, at the highest conductance values or highest X-ray flux, a saturation is found.

**Figure 2:**
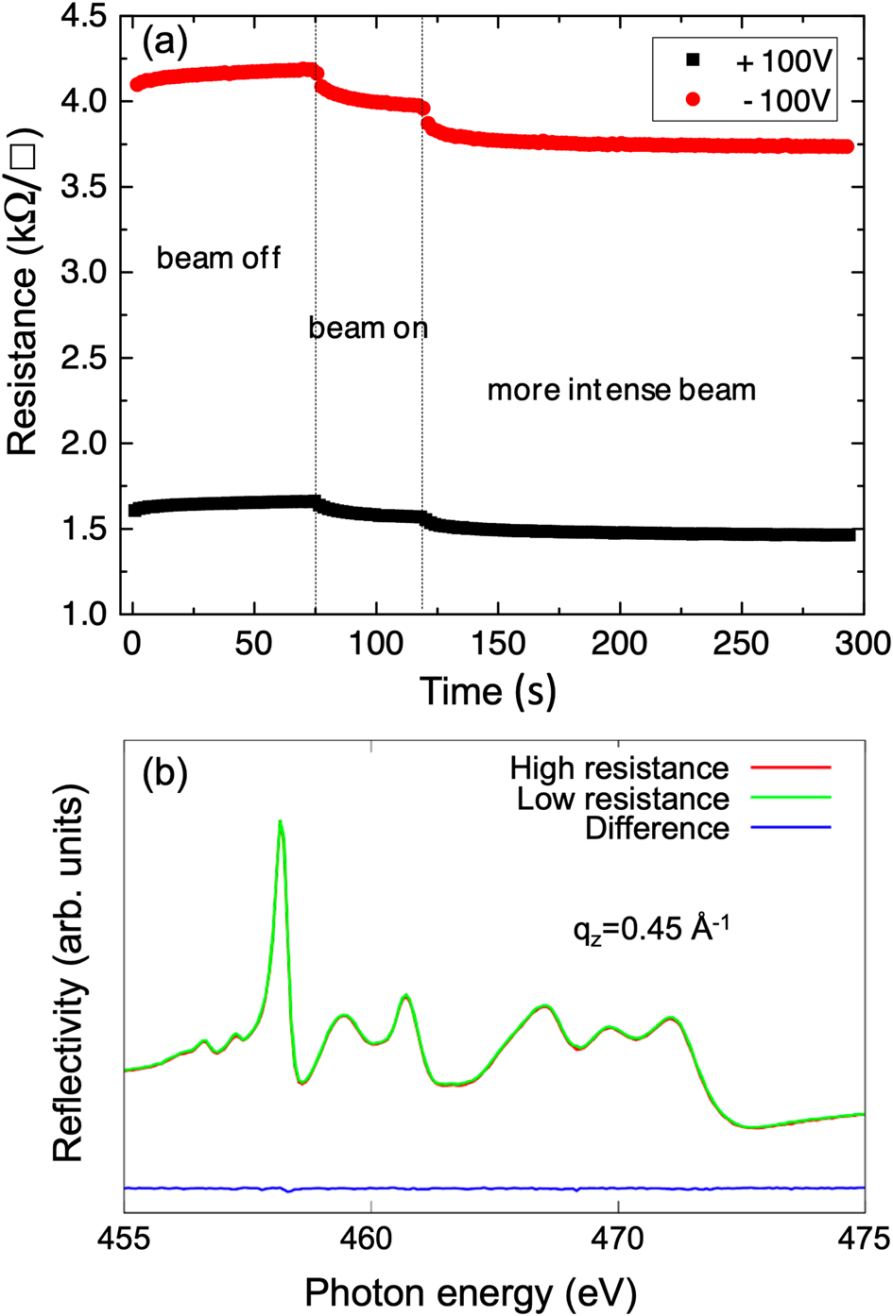
(**a**) Variation of $R_{sheet}$ upon alternating $V_G$ between + 100 V and - 100 V with and without X-ray irradiation. Even under X-ray illumination a large change in the sample averaged $R_{sheet}$ is induced by $V_G$ in a reproducible way. (**b**) Resonant reflectivity at the Ti $L_{2,3}$ edge for a fixed scattering vector $q_z$ = 0.45 Å$^{-1}$ in the nominal low-resistance and high-resistance state (see text) measured at T=11 K. No difference in the spectrum is observed. The large $q_z$ vector was chosen in order to increase the spatial resolution.

Interestingly, starting from the irradiated low-resistance state, we observed that applying $V_G=+100$ V and then $V_G=-100$ V ($\pm 2$ kV/cm) recovers a high-resistance state, even under continued X-ray illumination. This is illustrated in Fig. 2(a), where the effect of both the X-ray irradiation and switching $V_G$ is evident. More precisely, the relative variation of the sheet resistance between the two states with high ($\rho _H$) and the low ($\rho _L$) sheet resistance just after this voltage switch was $(\rho _H-\rho _L)/\rho _H \simeq 0.6$. This implies that the change of the *sample-averaged* $R_{sheet}$ due to the back gate voltage proceeds on a significantly faster time scale than that caused by X-ray irradiation. The latter evolves according to Fig 1(b) on the timescale of minutes.

To characterize the effect of the X-ray irradiation on the 2DEL further, we therefore applied the following measurement protocol: for each photon energy of a fixed-q scan, we collected a single data point in the high-resistance state with a short irradiation of a few seconds. Then we switched the sample to the low-resistance state by applying $V_G=+100$ V and collected a second RXR data point in this condition. We then moved to the next photon energy and flipped back to the high-resistance state using the protocol described above. Fig. 2(b) shows the result of this experiment performed at T=11 K. No difference between the RXR at negative and positive $V_G$ has been detected within the errors of our measurement. Even after applying a large gate voltage up to $\pm ~500\,\text {V} (\pm 10$ kV/cm), no significant change in the reflectivity pattern could be discerned. We also reduced the photon flux on the sample in order to mitigate the irradiation effect on the 2DEL, but in all cases the RXR at positive and negative $V_G$ remained identical within the experimental error. Note that such a fixed-q scan at resonance is extremely sensitive to changes in the electronic depth profile related to the Ti 3*d*-electrons. We estimate (cf. discussion below) that the $V_G$-induced change of the Ti 3*d* charge in the 2DEL located within the beam spot is well below 0.1 $e^-$ per unit cell (UC).

To understand these results it is important to realize that the sample surface area in this experiment was about $\sim 5~\text {mm}^2$, whereas the X-ray beam was focused onto an area of about $50\times 100\,\upmu \text {m}^2$. The sample volumes probed by the electrical sheet resistance and RXR were therefore *not* the same. We also observed that even if the focused X-ray beam is located beside the sample, its halo is still strong enough to cause the irradiation effect. We confirmed this by moving the beam on and off the sample while recording the sheet resistance change.

These observations indicate that $R_{sheet}$, which averages over a large sample area, might not be representative of what happens locally within the focused X-ray beam spot. The observed behavior – in particular the RXR data in Fig. 2 – suggests that the electronic properties of the 2DEL within the beam spot reach a beam-saturated low-resistance configuration on a time scale shorter than 1 s, i.e. much faster than the dynamics shown in Fig. 1(b). In any case, the present results demonstrate that soft X-ray irradiation modifies the electronic properties of the LAO/STO, driving the system into a low-resistance state. Caution is therefore required when drawing conclusions from such measurements about the unperturbed system. For the RXR-studies presented in the following, the above means that we are in fact always analyzing the irradiated low-resistance state of the LAO/STO interface.

### Resonant X-ray reflectivity

The analysis of the RXR data was done using the atomic slice approach described in Ref.^20^. In this approach, the crystal structure of the LAO/STO-sample is modeled in terms of a stacking of atomic slices along the (001) film normal. The atomic slices used for the present analysis therefore correspond to the atomic SrO, TiO$_2$, LaO and AlO$_2$ planes of STO and LAO. Their thickness and stacking has been defined according to precise crystallographic data for LAO/STO heterostructures^21,22^. For each of the atomic slices an energy dependent index of refraction $n(\omega )$ was then calculated using the $f(\omega )$ of the elements and their concentrations^20,23,24^. Regarding the termination of substrate and film, the LAO/STO interface in the models was fixed to be TiO$_2$-LaO, since the films were grown on TiO$_2$-terminated substrates, whereas no preference for the surface termination of LAO was assumed.

We parametrized the system in terms of the thickness of the LAO film, the thickness of the adsorbates (modeled by carbon) as well as the roughness at the interface and at the surface. For the non-resonant O-, Sr- and Al-sites we took tabulated values of the scattering amplitudes^25^, whereas for La we used the $f(\omega )$ obtained in Ref.^20^. Ti required a different approach, because at the Ti $L_{2,3}$-edge the different $f(\omega )$ for the nonequivalent Ti-positions in the heterostructure can neither be reliably deduced from experimental data nor can they be taken from tabulated values. This applies in particular to the Ti-sites close to the interface. We therefore modeled the $f(\omega )$ of Ti employing multiplet calculations, which were done using the program *Quanty*^27^. The electron-electron interaction Slater integrals ($F_{2pd} = 3.628$ eV, $F_{2dd}=5.896$ eV, $F_{4dd}=3.704$ eV, $G_{1pd}=3.153$ eV, $G_{3pd}=1.792$ eV) were estimated from density functional theory and slightly modified to improve the agreement with the XAS measurements. The Ti 2*p* spin–orbit coupling was set to $\zeta_{2p}=3.71\,{\text{eV}}$, whereas the spin-orbit coupling of the Ti *d* electrons was set to zero after noticing that values of $\zeta_{3d} < 10\hbox { meV}$ leave the absorption spectra almost unchanged^26^. The cubic component of the crystal field was set to $10Dq = 2.2$ eV for both $\hbox {Ti}^{3+}$ and $\hbox {Ti}^{4+}$ and the energy dependent lifetime broadening was chosen as to match the experimental X-ray absorption for $\hbox {Ti}^{4+}$ and the X-ray reflectivity for $\hbox {Ti}^{3+}$ in the fits.

The 2DEL is modeled in terms of additional electrons that populate the otherwise empty Ti 3*d*-orbitals near the interface. This corresponds to partially reducing the interface $\hbox {Ti}^{4+}$ ions to $\hbox {Ti}^{3+}$, as observed in earlier reports^28,29^. We therefore included $\hbox {Ti}^{3+}$ in the interface region, whereas the Ti deep inside STO was assumed to be only of $\hbox {Ti}^{4+}$ character. Earlier studies already established that a structural distortion within STO close to the interface causes an additional energy splitting of the Ti 3*d*-orbitals^30–32^. To include this effect into the present model, the energy splitting in the $e_g$ and the $t_{2g}$ sector was included as a model parameter for the two TiO$_2$ layers closest to the interface, which provided a good trade-off between model accuracy and reliability of the fit convergence.

**Figure 3:**
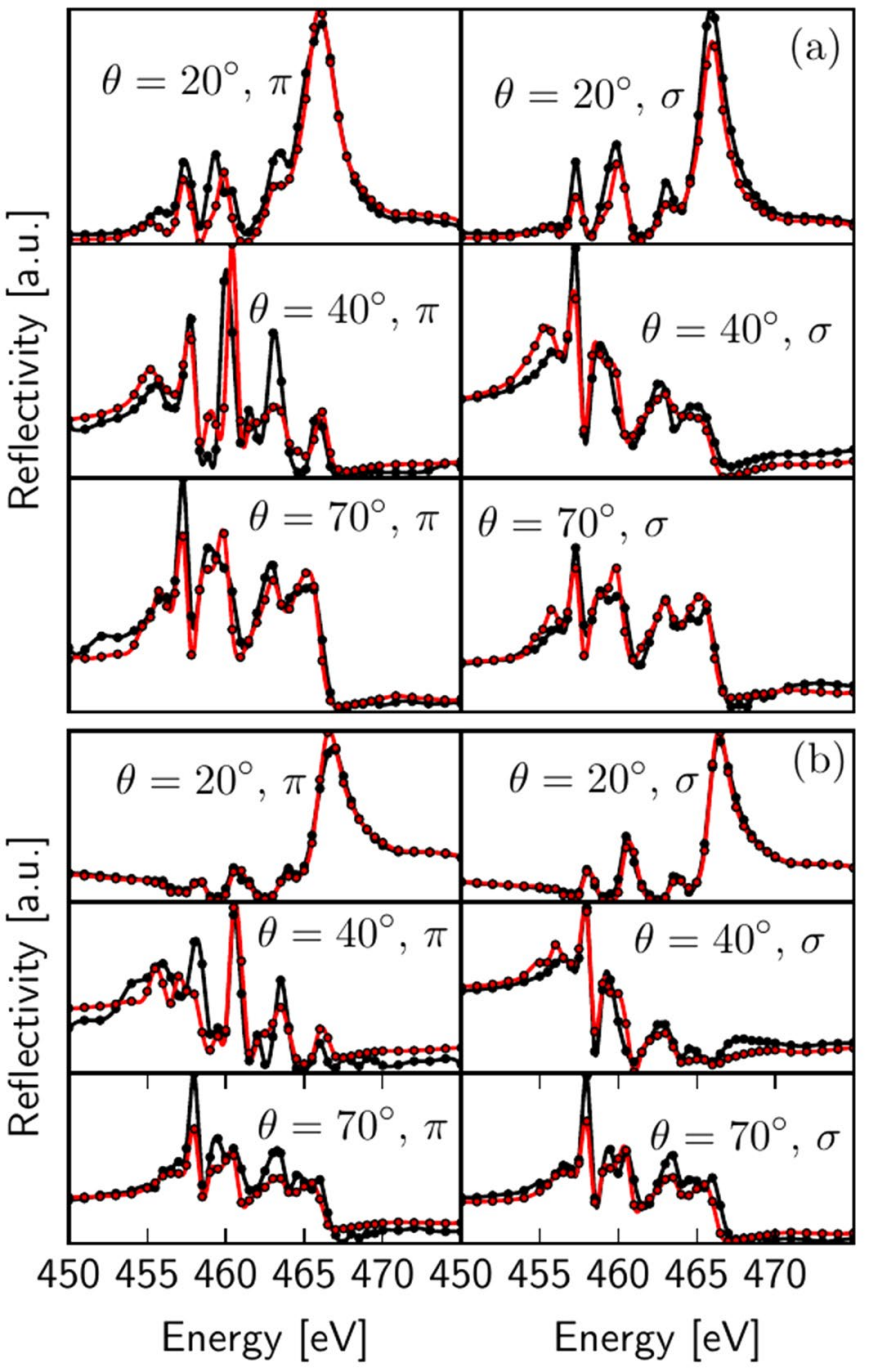
Ti $L_{2,3}$ resonant X-ray reflectivities measured at (**a**) room temperature and (**b**) T=11 K. The curves show energy dependent specular RXR-intensities for a fixed incident angle $\theta$ and for incident $\sigma$ and $\pi$ polarization. The experimental data is shown in black and the calculated reflectivity in red.

The atomic slice model was then fitted to the experimental RXR data, such that all of the approximately 12000 data points for a given temperature were fitted simultaneously. Importantly, the analysis included a fitting of the $\hbox {Ti}^{3+}$ content and its depth profile, which corresponds to the density $\rho _{t_{2g}}$ of the Ti $t_{2g}$-electrons. These electrons contribute to the conducting 2DEL at the LAO/STO interface. Figure 3 depicts some representative experimental RXR data (black) obtained for room temperature (a) and T=11 K (b) together with the corresponding fit results (red). Just like the fixed-q scans described earlier, these fixed-$\Theta$ scans are very sensitive to the depth profile of the Ti 3*d* electron system and enable detection of subtle variations as a function of temperature. Already visual inspection of the experimental data in Fig. 3 reveals clear temperature dependences, uncovering directly a temperature dependence of Ti 3*d* depth profile.

**Figure 4:**
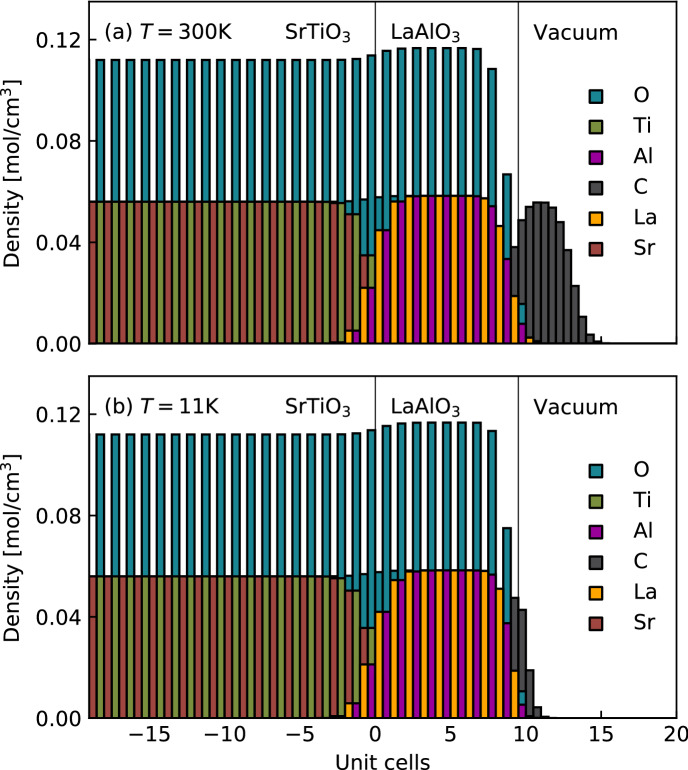
Densities of the different elements as obtained from RXR. Each vertical block represents the density of one atomic species within an atomic slice at a given distance from the interface, which is located at 0 UC on the abscissa. Note that the blocks of different species are overlapping, as the blocks always reach from 0 mol/cm$^3$ to the actual density. For instance, for a SrO-layer only the Sr-block is visible, because it is on top of the O-block behind it. Models for room temperature (**a**) and at T = 11 K (**b**) are shown. The temperature dependence of the C-contamination layer can be attributed changes in the vacuum conditions upon cooling.

The detailed depth profiles at both temperatures are disclosed by the analysis in terms of the above atomic slice model. The corresponding monolayer-resolved density distributions are displayed in Fig. 4. In this figure, each vertical block represents one lattice plane of the LAO/STO heterostructure. The analysis reveals that the interfaces of our FEDs are sharp, with an interface roughness $\sigma _i=(1\pm 0.1)$ UC and a surface roughness $\sigma _s=(0.7\pm 0.1)$ UC. Note that the interface roughness, though small in absolute values, is larger than the surface roughness. If the films were locally ideal and the roughness were just the result of steps in the substrate, one would instead expect the surface to be at least as rough as the interface. $\sigma _i > \sigma _s$ therefore indicates some degree of atomic intermixing across the interface in accordance with previous reports^28,33^.

**Figure 5:**
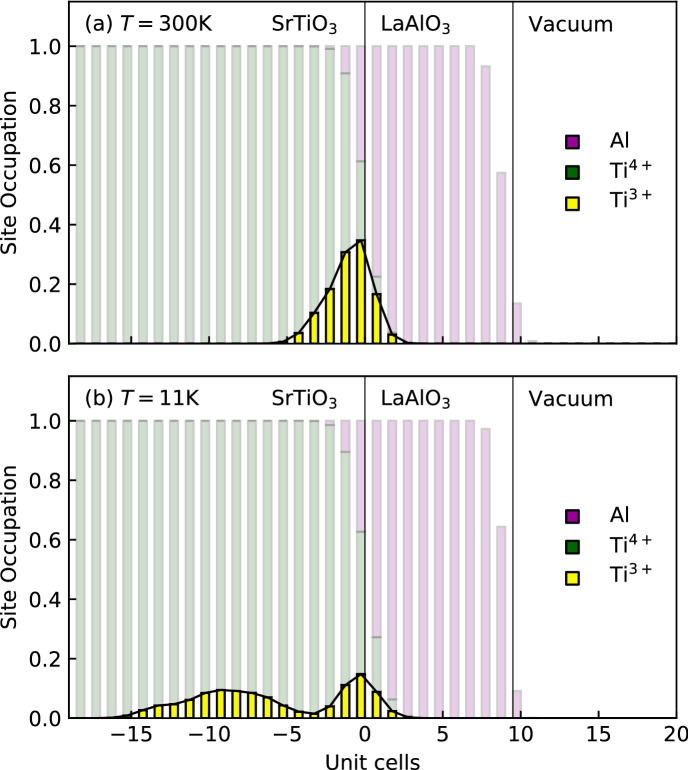
Temperature dependent electronic depth profile of $\hbox {Ti}^{3+}$ as obtained from the RXR-analysis. In contrast to Fig. 4, the bars represent the site occupation and the bars for different species are plotted on top of each other in the vertical direction so that they add up to the total site occupation in a given layer. For the $\hbox {Ti}^{3+}$, the site occupation corresponds to the average occupation of the of the Ti 3*d*-orbitals. The appearance of $\hbox {Ti}^{3+}$ in the LAO-layer indicates a small level of Al-Ti interdiffusion and/or the presence of TiO$_2$-terraces in a nominal AlO$_2$-layer. At low temperatures, a pronounced bimodal depth profile appears.

Figure 4 also shows the depth profiles obtained for the $\hbox {Ti}^{3+}$ species, which are again presented separately in Fig. 5. As can be observed in these figures, the $\hbox {Ti}^{3+}$depth profile is strongly temperature dependent. At room temperature the corresponding charge, i.e. $\rho _{t_{2g}}$, is located close to the LAO/STO interface and extends down to about 4 UC into the STO substrate. Upon lowering the temperature to 11 K, $\rho _{t_{2g}}$ penetrates much deeper into STO, reaching a thickness of $\sim 15-16$ UC. This is in very good agreement with earlier studies^14–17,28^. However, the monolayer depth-resolution of the present study reveals an additional and very surprising feature of $\rho _{t_{2g}}$ at low temperature: it is split into two well separated parts within the STO substrate. As shown in Fig. 5 (in blue), there is a minimum of the electronic density at about 4 UC from the interface. Hence, part of the Ti $t_{2g}$-charge is concentrated close to the LAO/STO interface and another part resides deeper inside the STO.

Using the $\hbox {Ti}^{3+}$ distributions shown in Fig. 5, we have calculated the integrated $t_{2g}$-electron densities for both temperatures. For both RT and 11 K we find a total $t_{2g}$-electron density of $(1.2 \pm 0.15)$ e$^-$ per 2D UC. This means that, although the charge is spatially redistributed upon changing the temperature, the detected amount of additional $t_{2g}$-charge at the interface remains constant within the experimental uncertainties. The total $t_{2g}$-electron density of about 1.2 e$^-$ per 2D UC obtained by RXR is, however, larger than the 0.5 e$^-$ per 2D UC value predicted by the electronic reconstruction scenario. As will become clear below, we argue that the additional charge carriers detected by RXR are due to oxygen vacancies.

**Figure 6:**
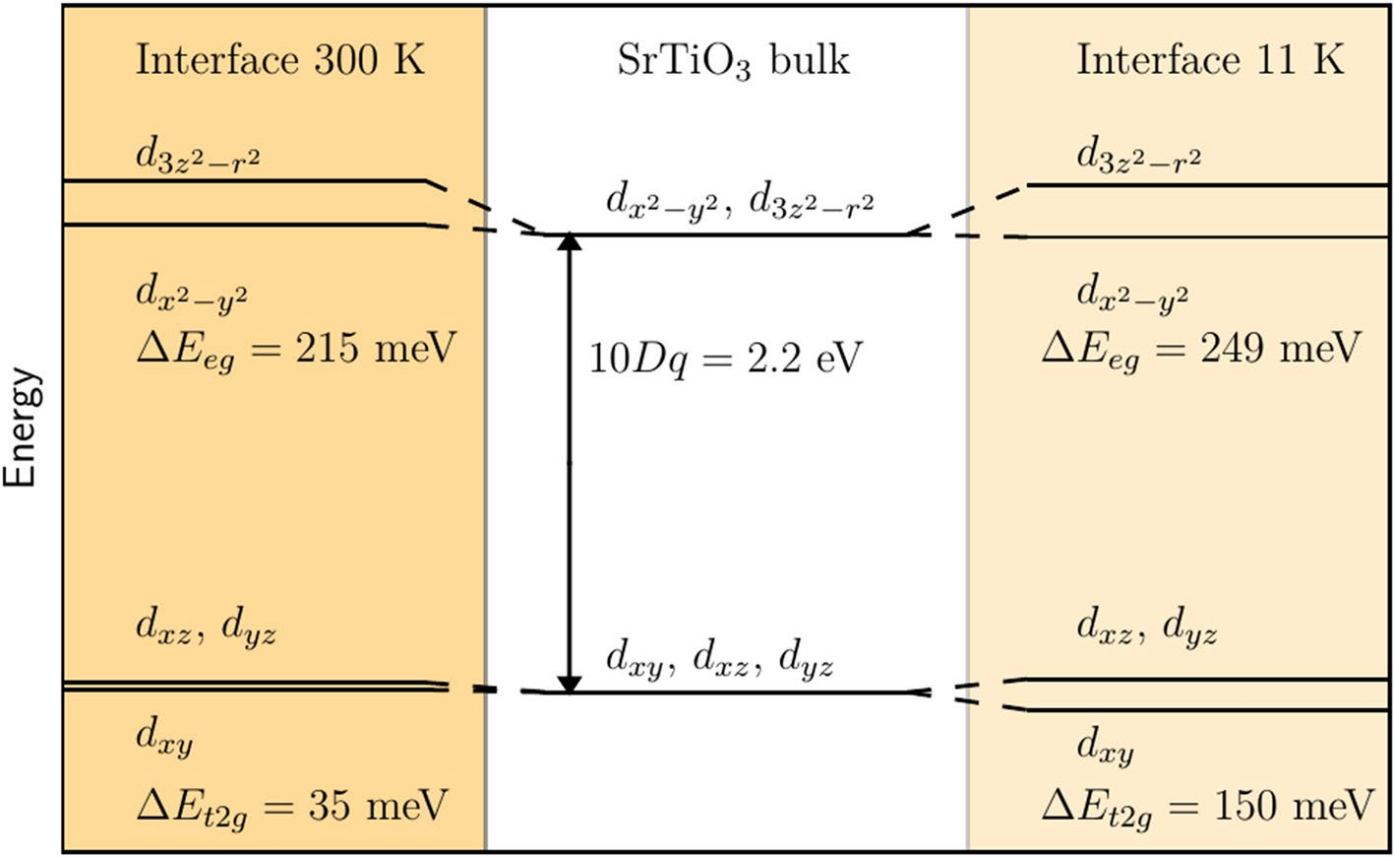
Energy splitting of the Ti $t_{2g}$ and $e_g$ orbitals within the two TiO$_2$-layers next to the interface for RT (left) and 11 K (right). The value of 10*Dq* for the bulk-like Ti was kept fixed.

The analysis of the RXR data also enables to extract depth-resolved information about the Ti 3*d*-orbitals. The present analysis yields an energy splitting of the $t_{2g}$ and $e_g$ orbitals of Ti that is qualitatively consistent with the orbital reconstructions reported earlier^30–32^. More in detail, for the $t_{2g}$ orbitals we obtain that the $d_{xy}$ is lower in energy than the degenerate $d_{xz}/d_{yz}$ orbitals. Likewise, the $d_{x^2-y^2}$ is found to be below the $d_{3z^2-r^2}$ in the $e_g$ sector. The value for the energy splitting obtained within the present model, are given in Fig. 6. The splitting of the $t_{2g}$ orbitals at RT is consistent with previous X-ray absorption studies (XAS)^30–32^, whereas the splitting of the $e_g$ levels is significantly larger. One possible reason for this discrepancy may lie in the depth-averaging of XAS, which differs from our depth-resolved approach. In any case, a larger splitting of the $e_g$ orbitals makes sense, since these orbitals are much more strongly influenced by geometrical variations of the ligand coordination, i.e., changes of bond distances and bond angles. The temperature dependent orbital energy splitting at the interface indeed agrees qualitatively with the observed increased polar structural distortion upon cooling, which develops within the STO close to the interface. We note that the typical energy steps of 50 meV used for the present RXR measurements cause uncertainties for the quantitative values of the orbital energy splitting. This can be improved by using finer energy steps and an extended, less constrained crystal field analysis. This more extensive approach is beyond the scope of the present study and therefore subject of another dedicated and comprehensive publication on that matter^34^.

## Discussion and conclusion

By combining transport measurements and RXR into a single experiment, we have shown that the electronic properties of STO/LAO change upon irradiation with soft X-rays. This observation is in general agreement with previous studies^3,35–43^, but was not systematically addressed so far by combining field effect, electrical transport, and spectroscopic measurements. Our studies of the irradiation effect indeed imply that the RXR-measurements performed in this work always probe the irradiated low-resistance state.

Notwithstanding, our RXR-study reveals subtle but important electronic changes at the LAO/STO interface as a function of temperature: Firstly, we find the interface charge distribution of the Ti t$_{2g}$-electrons ($\rho _{t_{2g}}$) to be strongly temperature dependent. This confirms previous studies employing conductive tip atomic force microscopy^15^, time resolved photo luminescence spectroscopy^44^ and hard X-ray photoemission spectroscopy^28^, which, however, did not provide the monolayer depth-resolution achieved here. The temperature dependence of the 2DEL at the LAO/STO interface has mostly been discussed in terms of the strong increase of the dielectric constant $\epsilon$ of STO upon cooling^14,16,17,45^. Since the temperature trends obtained by RXR and various other experimental techniques agree, we conclude that the spreading of $\rho _{t_{2g}}$ deeper into STO is not driven by the X-ray irradiation, but an intrinsic property of STO/LAO heterostructures.

Secondly—and more importantly—the monolayer depth-resolution of RXR yields a non-monotonous, bimodal depth profile of $\rho _{t_{2g}}$ at low temperatures. The resulting splitting into two separate parts is surprising and has not been observed in previous experiments. However, a very similar bimodal depth profile has been found in an earlier density functional theory (DFT) study by Li et al.^46^. By modelling oxygen vacancies in the LAO-layer as well as structural relaxations in the interface region, the authors obtained a charge density distribution very similar to the experimental one presented in Fig. 5 for $\text {T}=11$ K. Note that, since DFT calculations are ground state calculations, they should be compared to our low-temperature results only. This agreement between experiment and theory supports the claim that the unexpected bimodal interface charge distribution found in the present experiment is indeed intrinsic. It also indicates the presence of oxygen vacancies in our samples. This is perfectly in line with the high total $t_{2g}$-density of about 1.2 e$^-$ per 2D UC found in the present RXR-analysis, since oxygen vacancies act as electron donors. The DFT study by Li *et al.* finds that oxygen vacancies are more stable inside the LAO. However, oxygen vacancies can also occur inside the STO^47,48^. While our RXR-analysis does not allow determining the location of the oxygen vacancies, it is important to point out that a recent RXR study by some of us indeed identifies oxygen vacancies as the origin for the high $t_{2g}$-density^34^. The extra charge of about 0.7 e$^-$ per 2D UC on top of the 0.5 e$^-$ per 2D UC from polar catastrophe is therefore not due to the irradiation with soft X-rays.

We argue that the origin of the temperature dependence of $\rho _{t_{2g}}$ lies in the complex interplay between lattice and electronic degrees of freedom, which in turn couples the 2DEL to the strongly temperature-dependent STO dielectric function^49,50^. This is indeed strongly supported by the DFT results of Li *et al.*, who obtained a similar bimodal distribution by including a relaxation of the lattice^46^. Other lattice-related phenomena at the STO/LAO interface, such as the reported polar distortions^29,51,52^ and the strong electron-phonon coupling^53^, further highlight the active role of the lattice for the formation of the 2DEL. Temperature-driven changes in the lattice structure and in its dynamics therefore provide at least a qualitative route to explain the temperature evolution of $\rho _{t_{2g}}$. But still, the origin of the temperature dependencies observed here, and in particular the formation of the bimodal interface charge distribution at low temperatures, remains to be understood in detail. Note that the present RXR-measurements do not allow to draw conclusions about the mobility of the t$_{2g}$-electrons in the two different regions. While the electrons close to the interface are expected to contribute to the formation of a conducting 2DEL, the mobility of the electrons deeper inside the STO remains to be determined in detail.

To conclude, the present RXR study underlines the complexity of the LAO/STO interface and the effect of intense photon beams on the 2DEL properties. Our results provide a robust and detailed experimental confirmation of a large temperature dependence of the 2DEL depth profile and, moreover, reveal a splitting of the 2DEL into two parts at low temperatures. The redistribution of charge within STO is relevant to the interpretation of temperature dependent transport measurements, since charge carriers at different depth typically have different properties and, in particular, may have very different mobilities. The present results therefore contribute to developing a thorough understanding of complex transport properties in STO/LAO devices and their evolution as a function of temperature.

## Methods

For the present study, a 10 unit cells (UC) thick $\hbox {LaAlO}_3$ thin film was grown on a TiO$_2$-terminated STO substrate by means of RHEED-assisted pulsed laser deposition at $750^\circ C$ in an oxygen partial pressure of $5\times 10^{-2}$ mbar. Target to substrate distance was fixed to 40 mm and the laser deposition rate to 1 Hz. The charge carrier density of the 2DEL was characterized by means of Hall effect measurements, yielding a value of $4\times 10^{13}$ cm$^{-2}$ at room temperature, which corresponds to 0.1 e$^-$ per 2D UC and is in line with previously reported values of properly oxidized samples. This relatively small carrier density found in Hall measurements is commonly attributed to the fact that not all electrons have the same mobility^54,55^. The present experiments were carried out at the UE46-PGM1 beamline of the BESSY II storage ring of the Helmholtz Zentrum Berlin (HZB), using a two-circle XUV-diffractometer. To determine the depth profile of the Ti 3*d* states across the LAO/STO interface at different $V_G$, we have carried out simultaneous RXR and sheet resistance measurements on LAO/STO FEDs.
